# A double-blinded, placebo-controlled, randomized study to evaluate the efficacy of perioperative dextromethorphan compared to placebo for the treatment of postoperative pain: a study protocol

**DOI:** 10.1186/s13063-023-07240-0

**Published:** 2023-03-29

**Authors:** Ian A. Jones, Amit S. Piple, Pui Yuk Yan, Donald B. Longjohn, Paul K. Gilbert, Jay R. Lieberman, Gligor V. Gucev, Daniel A. Oakes, Christina E. Ratto, Alexander B. Christ, Nathanael D. Heckmann

**Affiliations:** 1 University of Washington, Anesthesiology & Pain Medicine, Seattle, USA; 2 Department of Orthopaedic Surgery, Keck Medicine of USC, 1520 San Pablo St Suite #2000, Los Angeles, CA 90033 USA; 3 Keck School of Medicine of USC, Los Angeles, USA

**Keywords:** Clinical trials, Dextromethorphan, Opioids, Pain control, Total knee arthroplasty, Postoperative pain control

## Abstract

**Background:**

Pain management is a critical component of comprehensive postsurgical care, as it influences patient safety and outcomes, and inadequate control has been associated with the development of chronic pain syndromes. Despite recent improvements, the management of postoperative pain following total knee arthroplasty (TKA) remains a challenge. The use of opioid-sparing, multimodal analgesic regimens has broad support, but there is a paucity of high-quality evidence regarding optimal postoperative protocols and novel approaches are needed. Dextromethorphan stands out among both well-studied and emerging pharmacological adjuncts for postoperative pain due its robust safety profile and unique pharmacology. The purpose of this study is to evaluate the efficacy of multi-dose dextromethorphan for postoperative pain control following TKA.

**Methods:**

This is a single-center, multi-dose, randomized, double-blinded, placebo-controlled trial. A total of 160 participants will be randomized 1:1 to receive either 60 mg oral dextromethorphan hydrobromide preoperatively, as well as 30 mg 8 h and 16 h postoperatively, or matching placebo. Outcome data will be obtained at baseline, during the first 48 h, and the first two follow-up visits. The primary outcome measure will be total opioid consumption at 24 h postoperatively. Secondary outcomes related to pain, function, and quality of life will be evaluated using standard pain scales, the Knee Injury and Osteoarthritis Outcome Score for Joint Replacement (KOOS, JR) questionnaire, the Patient-Reported Outcomes Measurement Information System (PROMIS-29) questionnaire, and clinical anchors.

**Discussion:**

This study has a number of strengths including adequate power, a randomized controlled design, and an evidence-based dosing schedule. As such, it will provide the most robust evidence to date on dextromethorphan utilization for postoperative pain control following TKA. Limitations include not obtaining serum samples for pharmacokinetic analysis and the single-center study design.

**Trial registration:**

This trial has been registered on the National Institute of Health’s ClinicalTrials.gov (NCT number: NCT05278494). Registered on March 14, 2022.

**Supplementary Information:**

The online version contains supplementary material available at 10.1186/s13063-023-07240-0.

## Background

### Introduction

Postoperative pain management is a critical component of comprehensive postsurgical patient care [1]. Postoperative pain not only affects patient well-being and satisfaction, but also can lead to deleterious events that can impact operative outcomes, including tachycardia, hyperventilation, decreased alveolar ventilation, poor wound healing, and insomnia [2]. There is also evidence that poorly controlled pain after surgery may elicit pathophysiologic neural alterations, including peripheral and central sensitization that can evolve into chronic pain syndromes [1].

While there have been notable improvements in the way postoperative pain is managed in the last decade, significant challenges remain, particularly with respect to total knee arthroplasty (TKA). For example, the range of pain scores reported following TKA is greater than most other elective surgeries [3]. Historically, opioids have been the preferred drugs of choice for pain management following TKA. However, excessive opioid administration is associated with significant side effects, including ventilatory depression, sedation, nausea, vomiting, pruritus, ileus, urinary retention, and constipation [1, 4]. Additionally, increasing opioid use within the early postoperative period following TKA is associated with a dose-dependent risk of periprosthetic joint infection and venous thromboembolic events [5, 6]. Moreover, liberal opiate utilization in healthcare has directly contributed to the opioid epidemic in the USA over the past 2 decades [7, 8]. In response, arthroplasty surgeons have significantly reduced opioid prescriptions in recent years to mitigate the risk of dependence [9].

A broad consensus has emerged in support of opioid-sparing, multimodal analgesic regimens [2]. The principal goal of multimodal analgesia is to target different steps of the pain pathway, resulting in more effective pain control with fewer side effects [10]. However, while multimodal protocols have consistently demonstrated improved pain control with less reliance on opioids, there is a lack of evidence regarding optimal postoperative protocols and pathways [11, 12].

Dextromethorphan stands out among both well-studied and emerging pharmacological adjuncts for postoperative pain due its robust safety profile and unique pharmacology. Dextromethorphan was first approved by the Food and Drug Administration (FDA) in 1958 and is currently one of the most common compounds found in over-the-counter antitussives [13]. As such, there is extensive data demonstrating that adverse drug reactions tend to be infrequent, short term, and usually mild [14]. Its clinical applicability is further supported by its wide therapeutic window, lack of major contraindications, and the fact that there are no special monitoring requirements for its use [15]. With respect to its unique pharmacology, dextromethorphan is structurally related to alkaloid opioids such as morphine [15], but does not interact with the mu receptor or produce typical opioid effects, including dependence [16]. It is also known to have many interactions with several different receptor sites, including activity as a low affinity antagonist at NMDA receptors [16].

### Clinical and preclinical data

After tissue injury, pain is transmitted via A-delta and C-sensory fibers to dorsal horn neurons, leading to hyperexcitability via activation of NMDA receptors [17–19]. The resulting effect causes both peripheral sensitization, which is a reduction in the threshold of nociceptive afferents, and central sensitization, which is an activity-dependent increase in the excitability of spinal neurons [20]. This process has been described as the “wind-up” phenomenon, which produces prolonged and more severe pain [18]. Dextromethorphan is thought to mitigate these effects by blocking NMDA receptors along the spinothalamic tract, reducing the threshold for pain transmission via the A-delta and C-sensory fibers [20]. However, it is also likely that additional pathways are involved. For example, recent studies have used nerve ligation animal models to show that dextromethorphan-mediated improvements in hyperalgesia may be related to the inhibition of glucocorticoid receptor-mediated neuroinflammation [21] and decreased expression of pTyr1336NR2B receptors [22].

In addition to the aforementioned preclinical data, there is also strong evidence that dextromethorphan has beneficial effects when used to treat pain in human subjects. Most notably, numerous randomized controlled trials have shown that dextromethorphan leads to reduced postoperative pain and opioid consumption. A 2016 meta-analysis investigating perioperative dextromethorphan as an adjunct for postoperative pain identified 40 studies, of which 21 were eligible for one or more comparisons [23]. In 848 patients from 14 trials, opioid consumption favored dextromethorphan (mean difference: − 10.51 mg intravenous morphine equivalents). Additionally, pain (as measured using 10-point visual analog scale) at 1, 4–6, and > 24 h also favored dextromethorphan (Mean difference: − 1.60, − 0.89, and − 0.92, respectively).

### Study overview

While numerous studies have demonstrated that dextromethorphan is safe and effective for the treatment of postoperative pain, no large randomized controlled trials have been published to date [23]. Moreover, the data on dextromethorphan is particularly limited with respect to its use in lower extremity surgery. Furthermore, even amongst studies that have been published, there is a high degree of heterogeneity both between and among existing trials, which limits clinical application. For example, several studies to date have included interventions with drastically different pain and recovery profiles, such as hernia repair and knee arthroscopy [24, 25].

This single-center, multi-dose, randomized, controlled, double-blind superiority study aims to determine if perioperative dextromethorphan is superior to placebo for postoperative pain in patients undergoing TKA. Qualified participants will be randomized 1:1 to receive either dextromethorphan or placebo before and after surgery. We hypothesize that dextromethorphan will significantly reduce postoperative opioid consumption at 24 h compared to placebo in patients undergoing TKA. Secondary outcomes related to pain, function, and quality of life will be evaluated using standard pain scales, such as visual analog scale (VAS) or equivalent numeric rating scale (NRS) for pain with and without movement, the Knee injury and Osteoarthritis Outcome Score for Joint Replacement (KOOS, JR) questionnaire, the Patient-Reported Outcomes Measurement Information System (PROMIS-29) questionnaire, and clinical anchors.

## Methods

### Participants, interventions, and outcomes

**Fig. 1:**
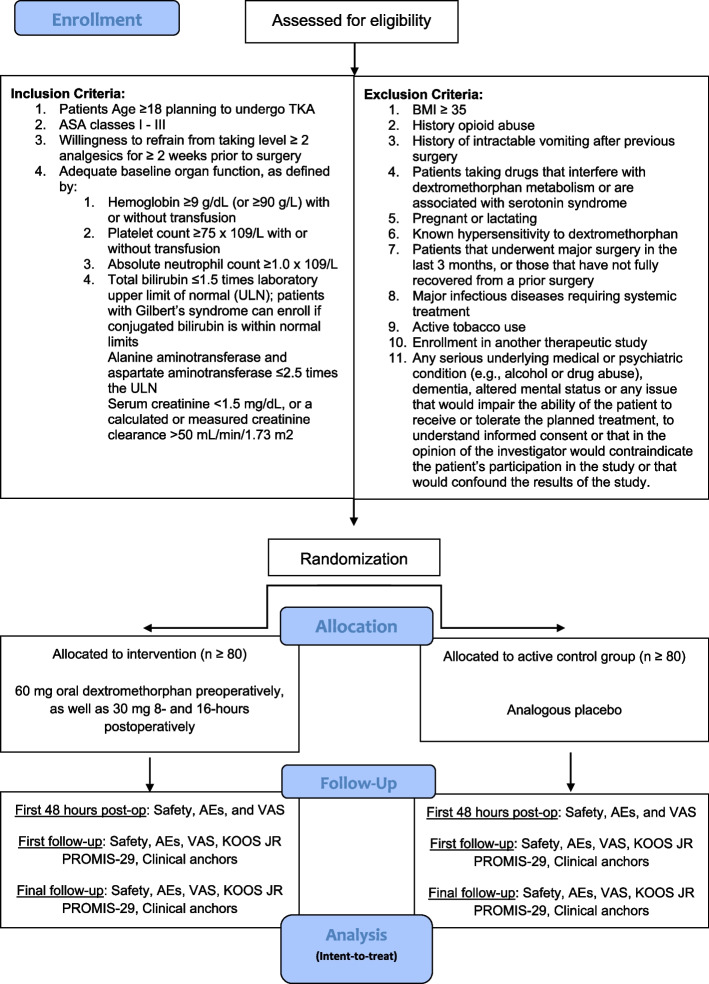
Study design flowchart. TKA = total knee arthroplasty; AEs = adverse events; VAS = visual analog scale; KOOS JR = Knee injury and Osteoarthritis Outcome Score for Joint Replacement; PROMIS = Patient-Reported Outcomes Measurement Information System

This is a single-center, multi-dose, randomized, double-blinded, placebo-controlled trial to evaluate the efficacy of perioperative dextromethorphan compared to placebo for postoperative pain in patients undergoing TKA. All standard of care interventions related to TKA are permitted during the trial. Patients aged 18 or older planning to undergo TKA will be screened for eligibility, as outlined in figure (Fig. 1). Those who meet inclusion/exclusion criteria and decide to participate will sign an informed consent prior to randomization. Patients randomized to the treatment group (*n* = 80) will receive 60 mg oral (pill) dextromethorphan hydrobromide preoperatively, as well as 30 mg 8 and 16 h postoperatively. Patients randomized to the control group (*n* = 80) will receive analogous oral placebo at the same time points.

A standardized, multimodal anesthetic protocol will be implemented. Preoperatively, patients will receive up to 400 mg celecoxib and 1000 mg acetaminophen. Intraoperative anesthetics will include spinal-epidural (15 mg bupivacaine) and propofol (75–100 µg/kg/min titrated to sedation). In the inpatient postoperative setting, oral oxycodone will be available as needed. All pain medications may be adjusted as indicated. All such adjustments will be logged and reported with the final published results.

Participants have the right to withdraw at any point during the course of the study without prejudice. Additionally, the investigator can discontinue participants at any time if medically necessary. Participants may also be withdrawn from the study for the following reasons: (1) concerns for patient safety, (2) noncompliance with study procedures or obligations, or (3) patient withdrawal of consent. In the event that a patient withdraws due to an adverse event thought to be at least possibly related to study treatment, they will be followed until the adverse event resolves or the patient is deemed medically stable by the Principal Investigator. All findings will be documented.

To ensure adherence to intervention protocols, the study drug or placebo will only be administered while the patient is hospitalized for their standard of care treatment. During this time, patients will receive extensive monitoring that is comparable to (or better than) what the standard patient receives when hospitalized for acute medical illness, including a nursing ratio that is equal to or better than 4:1. In addition, dedicated research staff and investigators will be available to ensure all interventions are administered appropriately.

The primary outcome will be the mean difference in postoperative opioid consumption (morphine equivalents) at 24 h. Secondary outcomes include mean subjective postoperative pain at 6, 12, 24, and 48 h after surgery, as measured using VAS (or equivalent NRS) with and without movement preoperatively; mean postoperative opioid consumption at 48 h; mean long-term pain and function, as measured using the pathology-specific KOOS, JR; mean overall subjective status, as measured using the 29 question PROMIS-29 questionnaire; and subjective improvement, as measured using clinical anchors for pain and overall improvement. Details regarding enrollment, interventions, assessments, visits, and when specific outcomes are obtained can be found in Table 1. In addition to efficacy outcomes, this study has several exploratory objectives, which may be reported independently from the main study results. The first of these exploratory aims is to assess whether baseline factors (e.g., poor physical function, anxiety, depression, fatigue, sleep disturbance) are associated with a differential response relationship to dextromethorphan. Second, we will plan to perform a retrospective review to assess whether dextromethorphan has an effect on the anesthetic course.

**Table 1:** Study calendar: Screening activities may be performed any day up to and including day 1 on study, but will always be performed prior to administration of study drug or placebo. POD1 = post-op day 1; KOOS JR = Knee injury and Osteoarthritis Outcome Score for Joint Replacement; PROMIS = Patient-Reported Outcomes Measurement Information System; 1 = As clinically indicated. A symptom-directed physical exam to be completed for AEs which are at least possibly related to study treatment

**Study procedure**	**Screening** **(day − 90)**	**Day 1**				**48 ± 4 h**	**Week 6 ± 3**	**Final** **(3–6 months)**
		**Pre-op**	**8 ± 4 h**	**16 ± 4 h**	**24 ± 4 h**			
**Screening/administrative**								
Sign main informed consent form	x							
Inclusion/exclusion criteria	x							
Medical history and demographics	x							
**Study drug administration**								
Administration of study drug or placebo		x	x	x				
**Safety assessments**								
Physical examination	x	x	x^1^	x^1^	x^1^	x^1^	x	x
Vital signs	x	x	x^1^	x^1^	x^1^	x^1^	x	x
Prior therapies	x							
Physical measurements	x							
Hematology	x							
Serum chemistry	x							
**Outcome evaluations**								
Post-op opioid use					x	x	x	x
VAS-pain with movement	x		x	x	x	x	x	x
VAS-pain at rest	x		x	x	x	x	x	x
KOOS JR	x						x	x
PROMIS-29	x						x	x
Clinical anchors							x	x
Adverse events			x	x	x	x	x	x

The analysis for the primary outcomes will be by intention-to-treat (ITT). All subjects will be analyzed according to randomization status, regardless of the actual treatment received, compliance with therapy, or adherence to the study protocol. For the primary study aim, we will compare the mean difference in postoperative opioid consumption between the placebo and control groups using generalized linear models (GLM) with a Gaussian family specification, assuming the data are normally distributed. If the data are not normally distributed, we will specify a more appropriate distribution based on a visual evaluation of the data using histograms. We will also use GLM to examine the relationship between treatment and outcome at each time point at which outcomes are measured. Last, for outcomes obtained at multiple time points, we will model the effect across all time points using a multi-level mixed effects model to account for repeated measures. Confounders will be defined as variables, which, when added to the model, alter the effect estimate by > 15%. Potential a priori confounders include body mass index (BMI), sex, age, race, and American Society of Anesthesia (ASA) grade. A *p*-value of < 0.05 will be considered statistically significant.

In addition to ITT analysis, per-protocol and sensitivity analyses will also be conducted. The per-protocol analysis will include only those participants who were protocol-adherent, as defined by their having received all assigned treatments and completed all follow-up visits. The sensitivity analyses will aim to identify potential sources of bias or uncertainty using various imputation methods.

There are 2 prior studies which can be used to estimate sample size for the proposed trial. The first was published by Wadhwa et al. in 2001 [26] and the second by Entezary et al. in 2013 [27]. Entezary et al. provides data that is consistent with a more conservative approach, as they used a much lower dose and investigated dextromethorphan for postoperative pain following arthroscopy–an intervention that results in relatively less pain than TKA. Using the smaller effect size from Entezary et al. (*μ* =  − 2.4, *σ* = 5.4) and standard methodology to compare the means using the T statistic with a non-centrality parameter [28, 29], a sample size of 160 patients (80 patients per group) would give 80% power to detect a difference in postoperative opioid consumption with a two-sided Type I error rate of 0.05. In contrast, using the same methodology for the study published by Wadhwa et al. yields a standardized effect size of 0.53, which corresponds to a sample size of 112 patients (56 patients per group). Given these estimates, we plan to recruit a total of 160 patients, which can accommodate 5–10% dropout. However, it should be noted that even if 5–10% of patients are lost to follow-up, the planned recruitment remains conservative. This is because the high doses used by Wadhwa et al. may not have necessarily increased the effect size in their study. In fact, it is possible that the larger dose used by Wadhwa et al. actually negatively impacted their effect size (by increasing adverse events), but these negative effects may have been offset by the fact that TKA is markedly more painful than knee arthroscopy.

### Assignment of interventions

Randomization will be conducted according to a central computerized randomization schedule (randomizer.at, from Medical University Graz, Austria; Url: http://www.randomizer.at/) with a 1:1 treatment allocation ratio and no stratification factors. The randomization method will be permuted blocks. Blocks of four were used to ensure equal numbers of subjects in each group. A separate investigator that is not involved in the recruitment, treatment, or assessment of participants will generate the allocation sequence and assign participants to the treatment or control group accordingly. Participants and investigators responsible for recruitment and treatment will be blinded. Treatment will be immediately and permanently discontinued and clinical staff will be unblinded if participants experience one or more of the following adverse events thought to be at least possibly related to study treatment: visual hallucinations, delusions, agitation, stupor, coma, respiratory depression, seizures, or any sign of serotonin syndrome.

## Data collection, management, and analysis

Demographics and survey data will be entered directly into the Patient IQ database (Additional file 1: Appendix A). Patient IQ has many built-in security features, including off-site backups, an audit trail, secure logins, de-identified data exports, and built-in filtering methods to ensure data quality. Survey answers (clinical outcome data) will be directly entered into the database by the participants themselves (via computer or mobile), which will improve data quality by removing the possibility of transfer errors. Documentation not included in the quantitative analysis of clinical outcomes will be recorded using traditional source documents (Additional file 2: Appendix B). These documents will be kept in the participant’s binder and locked in a secure location. Standard protocols to promote participant retention and ensure follow-up will be implemented. These include, but are not limited to, reminder notifications pushed to participants via the Patient IQ platform and direct correspondence from research staff.

Outcome instruments include standardized assessments of subjective pain (as measured using VAS or NRS 0 to 10), the KOOS, JR questionnaire, the PROMIS-29 questionnaire, and clinical anchors. KOOS, JR is a validated, short-form knee arthroplasty outcomes survey that combines pain, symptoms, and functional limitations into a single score representing overall “knee health” [30]. The PROMIS-29 questionnaire is a set of patient-centered measures that integrates functional limitations, pain interference/intensity, ability to fulfill desired social roles, anxiety/depression, sleep disturbance, and fatigue [30, 31]. PROMIS-29 has been shown to correlate with scales measuring similar constructs for patients with osteoarthritis [32] and, similar to KOOS, JR, is responsive in patients undergoing total joint arthroplasty [33]. Lastly, three clinical anchors (i.e., pain, physical function, and overall change) will be collected with patient-reported outcome (PRO) measures to determine the patient’s global impression of change and validate clinical significance when appropriate. The rationale for the collection and drafting of these anchors has been described at length previously [34–36].

### Monitoring

This is a single-site, investigator-initiated clinical trial and, as such, a formal data monitoring committee will not be utilized. However, the principal investigator, a study monitor, and the lead clinical research coordinator will regularly review study data for the occurrence of mild, moderate, and severe toxicity/adverse events. Mild toxicity/AEs include tachycardia and blurred vision. Moderate toxicities/AEs include visual hallucinations, delusions, stupor, and respiratory depression. Severe toxicity/AEs include coma, seizure, or symptoms concerning for serotonin syndrome. The study will be suspended if more than 1 out of the first 10 patients enrolled in the study group experience moderate or worse adverse event(s) thought to be at least possibly attributable to the investigational treatment.

All adverse events will be recorded in study source documents. Spontaneously reported adverse events will also be reported to the university IRB in accordance with federal and institutional regulations. All serious adverse events thought to be at least possibly related to the study treatment will be reviewed by the PI, a safety monitor (board-certified orthopedic surgeon not directly involved in clinical study activities), as well as lead study coordinator. Adverse events will be identified by regularly reviewing study data. Additionally, the Patient IQ database will automatically notify the independent clinical medical monitor and lead study coordinator when moderate or worse AEs reported. Subjects experiencing serious adverse events will be treated according to the standard of care and followed clinically until their health has returned to baseline status or until all parameters have returned to normal.

### Ethics and dissemination

Ethics approval was requested from the University of Southern California Health Science IRB on 20 March 2022. Continuing reviews and all protocol modifications will be submitted to the USC Health Science IRB through the University iStar System. Because this study involves “greater than minimal risk,” as defined in federal regulations 45 CFR 46.102 and 21 CFR 50.3, only designated clinical investigators will be allowed to obtain informed consent. Consent forms will be obtained prior to any study procedures and participants will be given a personal copy of the consent. Additionally, an informed consent comprehension assessment will be performed before randomization. There are no special provisions for ancillary or post-trial care or compensation for those who suffer harm from trial participation. However, participants will be informed prior to enrollment that they are responsible for unscheduled visits/interventions and are not giving up any legal rights by agreeing to participate in the study.

To protect participant confidentiality, participants will be assigned a subject identification number that will be used to record study-specific data. Only IRB-approved study personnel will have access to information linking subject identification numbers to identifiable information. Additionally, the link between study participants and their study ID will be destroyed in accordance with federal regulations when study activities are complete. The final trial dataset, once purged of all identifiable data, will be made available to all study personnel listed on the IRB protocol. This de-identified dataset will also be stored by the PI indefinitely.

The International Committee for Medical Journal Editors guidelines will be used to determine authorship eligibility for all publications resulting from this study. An abstract containing preliminary data may be submitted prior to formal completion of the trial. Once study activities have been completed, the results of the study, whether positive or negative, will be published and posted to clinicaltrials.gov. This information will also be shared directly with participants upon request. There is no intended use of professional writers, but The Keck.

Media Relations Team may assist in developing a press release if results prove impactful.

## Discussion

As discussed, there were only two prior studies that could be used when estimating the sample size for this trial. The first, which was published by Wadhwa’s group, is a dose escalation study, which looked at large-dose oral dextromethorphan as an adjunct to patient-controlled analgesia with morphine after knee reconstruction or replacement [26]. In the dose escalation portion of the trial, the maximum tolerated dose (MTD), which was defined as the largest dose not associated with severe side effects such as nausea with vomiting on more than one occasion, heavy sedation, or hallucinations, was determined to be 750 mg. Based on this data, it was initially decided that patients would receive 400 mg preoperatively followed by two 200 mg doses postoperatively. However, preoperative and postoperative dosing was reduced to 200 mg for all timepoints after frequent incidences of side effects such as severe rash, nausea, and vomiting were observed in patients after receiving the 400 mg dose. Nevertheless, at the conclusion of the trial, contingency analyses of the treatment (*n* = 34) and placebo (*n* = 22) groups demonstrated nausea to be the only side effect that was significantly increased. This finding prompted the authors to conclude that 200 mg given in 8-h intervals is the threshold of tolerance for patients administering morphine to themselves via PCA for postoperative pain relief. The Entezary et al. reference study was conducted on knee arthroscopic surgery candidates and dosed patients preoperatively with 1 mg/kg dextromethorphan (*n* = 54) or saline placebo (*n* = 58) [27]. Notably, at this comparatively lower dose, no side effects related to dextromethorphan were observed, which is consistent with the broader literature to date. Indeed, a 2016 meta-analysis investigating perioperative dextromethorphan as an adjunct for postoperative pain found that 10 studies reported either no side effects or a non-significant difference between groups. Of note, 5 of the included studies found fewer side effects in patients who had received dextromethorphan [23].

To date, Wadhwa et al. remains an outlier in that it is the only randomized controlled trial to report more frequent adverse events (specifically, transient nausea) in dextromethorphan-treated patients. Unsurprisingly, Wadhwa et al. is also an outlier with respect to dose, as the authors determined the minimum tolerated dose (MTD) to be 200 mg when administered every 8 h [26]. Most trials to date have used doses between 30 and 60 mg when administering dextromethorphan at multiple timepoints [23]. Multiple trials investigating dextromethorphan for postoperative pain have dosed patients with ≥ 60 mg [24, 25, 27, 37–42] or repeated dosing multiple times postoperatively [26, 39, 40, 43, 44], all of which have found improvement or no difference in the frequency of adverse events. Therefore, in the present trial, it was decided to dose patients at 60 mg preoperatively, followed by 30 mg at 8 and 16 h postoperatively, which correlates to the upper end of what has been used in most studies to date, while still being well-below the MTD reported by Wadhwa et al. This dose and duration between repeated doses is also consistent with known pharmacokinetics. For example, at the typical adult dose of 30 mg PO every 4 h, dextromethorphan has an onset of action of 15–30 min and a duration of action of 5–6 h [18]. Serum levels have been shown to peak approximately 2.4 h after a 2.5 mg/kg test dose [45], with a roughly 2-h delay in analgesic effect in relation to peak concentration [22].

The dosing of dextromethorphan is a non-trivial question, particularly in the context of inter-patient metabolic differences. Dextromethorphan undergoes rapid and extensive first-pass hepatic metabolism via cytochrome P450 2D6 (CYP2D6)-mediated O-demethylation to form the active metabolite, dextrorphan [16, 46]. The mean maximum plasma concentration of dextromethorphan and dextrorphan increases linearly with dose [47] and the elimination half-life is between 3 and 4 h [47]. Importantly however, four different metabolic groups have been described in the literature: ultrarapid, extensive, intermediate, and poor metabolizers. Roughly 90% of individuals are considered “extensive metabolizers,” while most of the remaining population are “poor metabolizers” [13]. The median half-life of dextromethorphan between these two groups differs widely, with the median dextromethorphan half-life in extensive metabolizers being 2.4 h and median half-life of poor metabolizers being 19.1 h [46].

This study includes precautions related to the development of serotonin syndrome. Dextromethorphan is known to inhibit serotonin reuptake and serotonin syndrome has been reported in patients who abuse the drug recreationally. However, it should be noted that only a handful of cases have been reported to date. Moreover, case reports that have linked dextromethorphan to serotonin syndrome have largely been limited to patients with exceptionally high serum concentrations and often occur in individuals taking other agents that potentiate excess serotonin in the body. For example, in one report, an 18-year-old male developed serotonin syndrome after recreational ingestion of Coricidin HBP. Confirmatory analysis demonstrated a dextromethorphan serum concentration of 930 ng/mL, and propofol infusion rapidly normalized his agitation, neuromuscular hyperactivity, and autonomic instability [48]. Another report described two cases of serotonin syndrome associated with supra-therapeutic doses of dextromethorphan in conjunction with a selective serotonin reuptake inhibitor (SSRI) [49]. Given the available data and exclusion of patients taking drugs that interfere with dextromethorphan metabolism or are associated with serotonin syndrome (e.g., monoamine oxidase [MAO] inhibitors, SRIs, amiodarone, and quinidine), the overall risk of serotonin syndrome in this trial has been determined by the investigators to be near negligible.

In 2010, the FDA approved the use of Nuedexta (dextromethorphan in combination with quinidine) for pseudobulbar affect, lending additional safety data for review. The combination therapy is based on the effect that low-dose quinidine has on increasing system concentration by reversibly inhibiting first-pass drug elimination [50]. During development, dose combinations of dextromethorphan/quinidine containing up to 6 times higher dextromethorphan and 12 times higher quinidine were studied. The most common adverse events were mild-to-moderate nausea, dizziness, and headache [51].

This study has several strengths, including adequate power, a randomized, controlled design, and an evidence-based dosing schedule. It will provide the most robust evidence to date on dextromethorphan for postoperative pain control following TKA. However, it is not without limitations. One major limitation is the lack of pharmacokinetic data. Given that there are inter-patient metabolic differences, there will be unaccounted for differences in how much drug each patient is exposed to over time. However, demonstrating clinical improvement despite this limitation has clinical utility, as widespread implementation of dextromethorphan is unlikely to be accompanied by patient-specific dose adjustments. More importantly, for the reasons discussed previously, dextromethorphan remains safe at the chosen dose level. This study effectively excludes the most profoundly sensitized chronic pain patients, as those with a history of opioid abuse and who are unwilling refrain from taking level ≥ 2 analgesics for ≥ 2 weeks prior to surgery are not allowed to participate. While this minimizes heterogeneity, it is not without drawbacks. Given the similarities between the pharmacological properties of ketamine and dextromethorphan, it is possible that chronic or sensitized pain patients are the most likely to show treatment effects. This was demonstrated by Loftus et al. [52] who found that intraoperative ketamine significantly reduced 48-h morphine consumption in patients taking high-dose preoperative opioids but had no effect on 48-h morphine consumption in patients consuming lower doses. At present, the aim of this study is to determine if the standard patient undergoing TKA stands to benefit from dextromethorphan. However, pending results of interim analysis, we recognize that it may be necessary to modify inclusion to allow for (or specifically target) patients with significant pain sensitization. Furthermore, the single-center design will limit the external validity of our findings.

## Supplementary Information

**Additional file 1: Appendix A.** Patient IQ datasheets.

**Additional file 2: Appendix B.** Source docs.

## Data Availability

Study materials will be available upon request once we have published our first original research paper from the data. Data will also be available at clinicaltrials.gov within 6 months of completion.

## Abbreviations

TKA: Total knee arthroplasty
KOOS Jr.: Knee Injury and Osteoarthritis Outcome Score for Joint Replacement
PROMIS-29: Patient-Reported Outcomes Measurement Information System
FDA: Food and Drug Administration
VAS: Visual analog scale
SSRI: Selective Serotonin Reuptake Inhibitor
NRS: Numeric Rating Scale
ITT: Intention to treat
GLM: Generalized linear model
ASA: American Society of Anesthesiologists
PRO: Patient-Reported Outcomes
BMI: Body mass index
MTD: Maximum tolerated dose
CYP2D6: Cytochrome P450 2D6
MAO: Monoamine oxidase
AE: Adverse event
