# Supplementary material for: A double-blinded, placebo-controlled, randomized study to evaluate the efficacy of perioperative dextromethorphan compared to placebo for the treatment of postoperative pain: a study protocol
Source: Trials. 2023 Mar 29;24:238. doi: 10.1186/s13063-023-07240-0 (PMC10061841; doi:10.1186/s13063-023-07240-0)
Supplement: Supplementary file 1 — Additional file 1: Appendix A. Patient IQ datasheets. [file 13063_2023_7240_MOESM1_ESM.pdf]

(/patients/48ed94de-65f9-40ab-b6f3-755a5c8e8a18/profiles) Piq Test **USC MRN:** 12345678 **DOB:** 02-22-1995 (27 yrs) **Sex:** female

Clinical Anchors

Intervention: --

Much Better

Form Fields

Clinical Anchors Questionnaire

Please rate the change since your knee surgery.

	MUCH WORSE	WORSE	ABOUT THE SAME	BETTER	MUCH BETTER
Please rate the change in your PAIN since your knee surgery.	○	○	○	○	○
Please rate the change in your PHYSICAL FUNCTION since your knee surgery.	○	○	○	○	○
Please rate the change OVERALL since your knee surgery.	○	○	○	○	○

Scroll for more options

Meta Data Fields

Submission Date
MM-DD-YYYY

MM-DD-YYYY

Cancel (/studies/1227/data#!clinical_anchors)

Save Save +

(/patients/48ed94de-65f9-40ab-b6f3-755a5c8e8a18/profiles) Piq Test **USC MRN:** 12345678 **DOB:** 02-22-1995 (27 yrs) **Sex:** female

Adverse Events

Intervention: **2022-09-22**

Form Fields

Submission Date
MM-DD-YYYY

MM-DD-YYYY

Adverse event(s)? (•) Yes ( ) No

Serious adverse event(s)? (•) Yes ( ) No

Adverse event(s) possibly related to study treatment? (•) Yes ( ) No

Please select all adverse events that apply:

- [ ] Nausea
- [ ] Vomiting
- [ ] Gastrointestinal discomfort
- [ ] Drowsiness
- [ ] Dizziness
- [ ] Nystagmus (involuntary eye movements)
- [ ] Rapid heart rate
- [ ] Blurred vision
- [ ] Visual hallucinations
- [ ] Delusions
- [ ] Stupor
- [ ] Respiratory depression
- [ ] Coma
- [ ] Seizures
- [ ] Metabolic syndromes
- [x] Other

Other description

Additional Comments:

Cancel (/studies/1227/data#!adverse_events)

Save Save +

(/patients/48ed94de-65f9-40ab-b6f3-755a5c8e8a18/profiles) Piq Test **USC MRN:** 12345678 **DOB:** 02-22-1995 (27 yrs) **Sex:** female

IP Log

Intervention: --

Form Fields

Submission Date MM-DD-YYYY	MM-DD-YYYY
IP taken at preop?	(●) Yes ( ) No
Time IP is taken at preop:	
Date IP is taken at preop: MM-DD-YYYY	MM-DD-YYYY
IP taken at 8 +/- 4 hrs?	(●) Yes ( ) No
Time IP is taken at 8 +/- 4 hrs:	
Date IP is taken at 8 +/- 4 hrs: MM-DD-YYYY	MM-DD-YYYY
IP taken at 16 +/- 2 hrs?	(●) Yes ( ) No
Time IP is taken at 16 +/- 2 hrs:	
Date IP is taken at 16 +/- 2 hrs: MM-DD-YYYY	MM-DD-YYYY
IP taken at 24 +/- 2 hrs?	(●) Yes ( ) No
Time IP is taken at 24 +/- 2 hrs:	
Date IP is taken at 24 +/- 2 hrs: MM-DD-YYYY	MM-DD-YYYY
IP taken at 48 +/- 4 hrs?	(●) Yes ( ) No
Time IP is taken at 48 +/- 4 hrs:	
Date IP is taken at 48 +/- 4 hrs: MM-DD-YYYY	MM-DD-YYYY

Cancel (/studies/1227/data#!ip_log)

Save Save +

(/patients/48ed94de-65f9-40ab-b6f3-755a5c8e8a18/profiles) Piq Test **USC MRN:** 12345678 **DOB:** 02-22-1995 (27 yrs) **Sex:** female

KOOS Jr

In Progress

KOOS JR (——)

Create Form

Intervention: **09-22-2022 (/study_data_events/10373054/edit?study_phase_id=10696)** **(Days)** None

Unlink (/study_data_events/10551767/unlink?study_phase_id=10664) Download (/study_data_events/10551767/edit.pdf?study_phase_id=10664)

Associated Task

KOOS Jr

Expired

Initially Sent:
09-21-2022

Archive (/patients/23617184/event/23617184) Expired: 10-21-2022

Due: 10-21-2022

-77 Days overdue

Form Fields

Instructions

This survey asks for your view about your knee. This information will help us keep track of how you feel about your knee and how well you are able to do your usual activities.

Stiffness* (required)

The following question concerns the amount of joint stiffness you have experienced during the last week in your knee. Stiffness is a sensation of restriction or slowness in the ease with which you move your knee joint.

	NONE	MILD	MODERATE	SEVERE	EXTREME
How severe is your knee stiffness after first wakening in the morning?	○	○	○	○	○

Scroll for more options

Pain* (required)

What amount of knee pain have you experienced the last week during the following activities?

	NONE	MILD	MODERATE	SEVERE	EXTREME
Twisting/pivoting on your knee	○	○	○	○	○
Straightening knee fully	○	○	○	○	○
Going up or down stairs	○	○	○	○	○
Standing upright	○	○	○	○	○

Scroll for more options

Function, daily living* (required)

The following questions concern your physical function. By this we mean your ability to move around and to look after yourself. For each of the following activities please indicate the degree of difficulty you have experienced in the last week due to your knee.

	NONE	MILD	MODERATE	SEVERE	EXTREME
Rising from sitting	○	○	○	○	○
Bending to floor/pick up an object	○	○	○	○	○

Scroll for more options

KOOS Jr

Meta Data Fields

Submission Date
MM-DD-YYYY
MM-DD-YYYY

Imported Form? ○ Yes ○ No

KOOS Jr Raw Score

Imported KOOS Jr Score

Use Imported KOOS Jr Score? ○ Yes ○ No

Imported Intervention Date
MM-DD-YYYY
Month Day Year

Imported Laterality ○ Right ○ Left

Cancel (/studies/1227/data#!koos_jr)

Save Save +

(/patients/48ed94de-65f9-40ab-b6f3-755a5c8e8a18/profiles) Piq Test **USC MRN:** 12345678 **DOB:** 02-22-1995 (27 yrs) **Sex:** female

Pre-Op Opioid Use

Intervention: **2022-09-22**

Form Fields

Which opioid medication(s) do you currently take? Please select all that apply.

- [x] Codeine (Panadeine, Nurofen Plus and Mersyndol)
- [x] Fentanyl Patch (Duragesic)
- [x] Hydrocodone (Hysingla, Vicodin, Lortab)
- [x] Hydromorphone (Dilaudid, Exalgo)
- [x] Methadone (Methadose, Dolophine)
- [x] Morphine (Arymo ER, AVINza, Kadian, Morphabond, MSiR, MS-Contin, Oramorph SR, Roxanol, RMS, Kadian, and oramorph SR)
- [x] Oxycodone (Dazidox, Eth-Oxydose, Oxaydo, OxyCONTIN, Oxydose, Oxyfast, Oxy IR, Roxicodone)
- [x] Oxymorphone (Numorphan, Opana)
- [x] Tapentadol (Nucynta)
- [x] Tramadol (Ultram, Conzip, Maxitram, Marol, Zydol, Zamadol, Tramulief, Tramquel)
- [x] Don't Know
- [x] Other

Codeine

How many tablets of codeine per day are you taking?

Fentanyl Patch

How many patches per day of fentanyl patch are you using?

Hydrocodone

What is the mg per tablet of hydrocodone you are prescribed?

How many tablets of hydrocodone per day are you taking?

Hydromorphone

What is the mg per tablet of hydromorphone you are prescribed?

How many tablets of hydromorphone per day are you taking?

Methadone

What is the mg per tablet of methadone you are prescribed?

How many tablets of methadone per day are you taking?

Morphine

What is the mg per tablet of morphine you are prescribed?

How many tablets of morphine per day are you taking?

Oxycodone

What is the mg per tablet of oxycodone you are prescribed?

How many tablets of oxycodone per day are you taking?

Oxymorphone

What is the mg per tablet of oxymorphone you are prescribed?

How many tablets of oxymorphone per day are you taking?

Tapentadol

What is the mg per tablet of tapentadol you are prescribed?

How many tablets of tapentadol per day are you taking?

Tramadol

What is the mg per tablet of tramadol you are prescribed?

How many tablets of tramadol per day are you taking?

Other Opioid:

Meta Data Fields

Submission Date
MM-DD-YYYY

MM-DD-YYYY

Cancel (/studies/1227/data#!pre-op_opioid_use)

Save | Save +

(/patients/48ed94de-65f9-40ab-b6f3-755a5c8e8a18/profiles) Piq Test **USC MRN:** 12345678 **DOB:** 02-22-1995 (27 yrs) **Sex:** female

PROMIS-29 V2.1 Never In Progress

PROMIS-29 (——)

Create Form

Intervention: **09-22-2022 (/study_data_events/10373054/edit?study_phase_id=10696)**
(Days) None

Unlink (/study_data_events/10551768/unlink?study_phase_id=10697) Download (/study_data_events/10551768/edit.pdf?study_phase_id=10697)

Associated Task
PROMIS-29

Expired

Initially Sent:
09-21-2022

Archive (/patients/23617185/event/23617185) Expired: 10-21-2022

Due: 10-21-2022

-77 Days overdue

Form Fields

Physical Function* (required)
Please respond to each question or statement by marking one box per row.

	WITHOUT ANY DIFFICULTY	WITH A LITTLE DIFFICULTY	WITH SOME DIFFICULTY	WITH MUCH DIFFICULTY	UNABLE TO DO
Are you able to do chores such as vacuuming or yard work?	○	○	○	○	○
Are you able to go up and down stairs at a normal pace?	○	○	○	○	○
Are you able to go for a walk of at least 15 minutes?	○	○	○	○	○
Are you able to run errands and shop?	○	○	○	○	○

Scroll for more options

Anxiety in the past 7 days...* (required)
Please respond to each question or statement by marking one box per row.

	NEVER	RARELY	SOMETIMES	OFTEN	ALWAYS
I felt fearful..	○	○	○	○	○
I found it hard to focus on anything other than my anxiety.	○	○	○	○	○
My worries overwhelmed me.	○	○	○	○	○
I felt uneasy.	○	○	○	○	○

Scroll for more options

Depression in the past 7 days....* (required)
Please respond to each question or statement by marking one box per row.

	NEVER	RARELY	SOMETIMES	OFTEN	ALWAYS
I felt worthless.	○	○	○	○	○
I felt helpless.	○	○	○	○	○
I felt depressed.	○	○	○	○	○
I felt hopeless.	○	○	○	○	○

Scroll for more options

Fatigue during the past 7 days...* (required)
Please respond to each question or statement by marking one box per row.

	NOT AT ALL	A LITTLE BIT	SOMEWHAT	QUITE A BIT	VERY MUCH
I feel fatigued.	○	○	○	○	○
I have trouble starting things because I am tired.	○	○	○	○	○
How run-down did you feel on average?	○	○	○	○	○
How fatigued were you on average?	○	○	○	○	○

Scroll for more options

Sleep Disturbance I* (required)
Please respond to each question or statement by marking one box per row.

	VERY POOR	POOR	FAIR	GOOD	VERY GOOD
In the past 7 days my sleep quality was...	○	○	○	○	○

Scroll for more options

Sleep Disturbance II* (required)
Please respond to each question or statement by marking one box per row.

	NOT AT ALL	A LITTLE BIT	SOMEWHAT	QUITE A LOT	VERY MUCH
In the past 7 days my sleep was refreshing...	○	○	○	○	○
In the past 7 days I had a problem with my sleep...	○	○	○	○	○
In the past 7 days I had difficulty falling asleep...	○	○	○	○	○

Scroll for more options

Ability to Participate in Social Roles and Activities* (required)
Please respond to each question or statement by marking one box per row.

	NEVER	RARELY	SOMETIMES	USUALLY	ALWAYS
I have trouble doing all of my regular leisure activities with others.	○	○	○	○	○
I have trouble doing all of the family activities that I want to do.	○	○	○	○	○
I have trouble doing all of my usual work (include work at home).	○	○	○	○	○
I have trouble doing all of the activities with friends that I want to do.	○	○	○	○	○

Scroll for more options

Pain Interference in the past 7 days...* (required)
Please respond to each question or statement by marking one box per row.

	NOT AT ALL	A LITTLE BIT	SOMEWHAT	QUITE A LOT	VERY MUCH
How much did pain interfere with your day to day activities?	○	○	○	○	○

	NOT AT ALL	A LITTLE BIT	SOMEWHAT	QUITE A LOT	VERY MUCH
How much did pain interfere with work around the home?	○	○	○	○	○
How much did pain interfere with your ability to participate in social activities?	○	○	○	○	○
How much did pain interfere with your household chores?	○	○	○	○	○

Scroll for more options

Pain Intensity* (required)

In the past 7 days how would you rate your pain on average?

0 10

Click to Use Slider

Never

0 1 2 3 4 5 6 7 8 9 10

No Pain Worst Pain Imaginable

Physical Function Raw Score

Anxiety Raw Score

Depression Raw Score

Fatigue Raw Score

Sleep Disturbance Raw Score

Ability to Participate in Social Roles and Activities Raw Scores

Pain Interference Raw Score

Physical Function T-Score

Anxiety T-score

Depression T-score

Fatigue T-score

Sleep Disturbance T-score

Ability to Participate in Social Roles and Activities T-score

Pain Interference T-score

Pain Intensity Score

Meta Data Fields

Submission Date
MM-DD-YYYY

MM-DD-YYYY

Historical Form Version

Cancel (/studies/1227/data#!promis-29_v2_1)

Save Save +

(/patients/48ed94de-65f9-40ab-b6f3-755a5c8e8a18/profiles) Piq Test **USC MRN:** 12345678 **DOB:** 02-22-1995 (27 yrs) **Sex:** female

Pain VAS (USC)

Intervention: --

Form Fields

What is the AVERAGE PAIN have you had in the LAST 24 HOURS?

What is the AVERAGE PAIN have you had in the LAST 24 HOURS?
0 — 100
Click to Use Slider
0 2 4 6 8 12 16 20 24 28 32 36 40 44 48 52 56 60 64 68 72 76 80 84 88 92 96 100
No pain — Worst pain imaginable

What is the AVERAGE PAIN you have AT REST (in bed, sitting, etc)?

What is the AVERAGE PAIN you have AT REST (in bed, sitting, etc)?
0 — 100
Click to Use Slider
0 2 4 6 8 12 16 20 24 28 32 36 40 44 48 52 56 60 64 68 72 76 80 84 88 92 96 100
No pain — Worst pain imaginable

What is the AVERAGE PAIN do you have when you are ACTIVE?

What is the AVERAGE PAIN do you have when you are ACTIVE?
0 — 100
Click to Use Slider
0 2 4 6 8 12 16 20 24 28 32 36 40 44 48 52 56 60 64 68 72 76 80 84 88 92 96 100
No pain — Worst pain imaginable

Meta Data Fields

Submission Date
MM-DD-YYYY

MM-DD-YYYY

Cancel (/studies/1227/data#!pain_vas_usc)

Save Save +
