# Supplementary material for: A double-blinded, placebo-controlled, randomized study to evaluate the efficacy of perioperative dextromethorphan compared to placebo for the treatment of postoperative pain: a study protocol
Source: Trials. 2023 Mar 29;24:238. doi: 10.1186/s13063-023-07240-0 (PMC10061841; doi:10.1186/s13063-023-07240-0)
Supplement: Supplementary file 2 — Additional file 2: Appendix B. Source docs. [file 13063_2023_7240_MOESM2_ESM.pdf]

Subject Initials | | | | **Subject ID** **Date:** / /

Day Month Year

Eligibility

1. **Inclusion Criteria**	**Yes**	**No**
1. Age ≥18 and planning to undergo TKA		
2. ASA classes I - III		
3. Willingness to refrain from taking level ≥ 2 analgesics for ≥ 2 weeks prior to surgery		
4. Adequate baseline organ function (by meeting all criteria below)<br>• Hemoglobin ≥9 g/dL<br>• Platelet Count ≥75 x 10^9/L (x10e3cells/mcL)<br>• Absolute Neutrophil Count ≥1.0 x 10^9/L (x10e3cells/mcL)<br>• Total Bilirubin ≤ 1.5 times ULN (Conjugated Gilbert's syndrome pt is ok)<br>• AST and ALT ≤ 2.5 times ULN<br>• Creatinine ≤ 1.5 mg/dL or Creatinine clearance >30mL/min/1.73 m^2		

2.

3. **Exclusion Criteria**	**Yes**	**No**
1. BMI ≥ 35		
2. History opioid abuse		
3. History of intractable vomiting after previous surgery		
4. Taking a drug that interferes with dextromethorphan metabolism or is associated with serotonin syndrome (e.g., MAO inhibitors, SRIs, amiodarone, and quinidine)		
5. Gelatin hypersensitivity		
6. Pregnant or lactating		

7. Known hypersensitivity (allergy) to dextromethorphan		
8. Underwent major surgery requiring general anesthesia in the last 3 months, or not fully recovered from a prior surgery (i.e., unhealed wound)		
9. Major infectious diseases (e.g. hepatitis, active viral, bacterial, systemic fungal infection) requiring systemic treatment		
10. Active tobacco use		
11. Enrollment in another therapeutic study		
12. Serious underlying medical or psychiatric condition that impair the ability of the patient to receive or tolerate the planned treatment, to understand informed consent or that in the opinion of the investigator would contraindicate the patient's participation in the study or that would confound the results of the study.		

/

Reviewed by: ____ Date: ____________________

Investigator signature

Subject Initials ☐☐☐ **Subject ID** **Date:** / /

Day Month Year

Subject Off Study

Date subject went off Study: //////   /   /

Day Month Year

Visits Completed (check all that apply):

/

☐ Screening ☐ Preoperative ☐ Postoperative

☐ 6-week follow-up ☐ 6-month follow-up ☐ Other**

SCHEDULED VISITS MISSED: ☐ Yes** ☐ No ☐ Not applicable

INDICATE OFF STUDY REASON: (select only one)

☐ Study Activities Completed

If the subject was withdrawn prior to completing the study (i.e. early withdrawal), select one of the following:

☐ Subject withdrawn – by Subject PRIOR to randomization**

☐ Subject withdrawn – by Subject AFTER randomization**

☐ Subject withdrawn – by PI PRIOR to randomization**

☐ Subject withdrawn – by PI AFTER randomization**

☐ Other**

If the subject was withdrawn, indicate specific reason(s): *(select all that apply)*

☐ Subject screen failed

☐ Subject did unable/unwilling to comply with study procedures

☐ Subject lost to follow-up

☐ Subject refused follow-up

☐ Due to adverse events or complications

☐ Other**/

**Additional explanation required: ____

____

FORM COMPLETED BY: ____

/
